# Incidence and Risk Factors of COVID-19 Vaccine Breakthrough Infections: A Prospective Cohort Study in Belgium

**DOI:** 10.3390/v14040802

**Published:** 2022-04-13

**Authors:** Veerle Stouten, Pierre Hubin, Freek Haarhuis, Joris A. F. van Loenhout, Matthieu Billuart, Ruben Brondeel, Toon Braeye, Herman Van Oyen, Chloé Wyndham-Thomas, Lucy Catteau

**Affiliations:** 1 Department of Epidemiology and Public Health, Sciensano, 1050 Brussels, Belgium; pierre.hubin@sciensano.be (P.H.); freekhaarhuis@gmail.com (F.H.); joris.vanloenhout@sciensano.be (J.A.F.v.L.); matthieu.billuart@sciensano.be (M.B.); ruben.brondeel@sciensano.be (R.B.); toon.braeye@sciensano.be (T.B.); herman.vanoyen@sciensano.be (H.V.O.); chloe.wyndham-thomas@outlook.com (C.W.-T.); lucy.catteau@sciensano.be (L.C.); 2 Department of Public Health and Primary Care, Ugent, 9000 Gent, Belgium

**Keywords:** vaccination, SARS-CoV-2, breakthrough infection, hybrid immunity, viral vector vaccines, mRNA vaccines, symptoms, COVID-19, mRNA booster vaccine

## Abstract

The objective of this study was to investigate the incidence and risk factors associated with COVID-19 vaccine breakthrough infections. We included all persons ≥18 years that had been fully vaccinated against COVID-19 for ≥14 days, between 1 February 2021 and 5 December 2021, in Belgium. The incidence of breakthrough infections (laboratory confirmed SARS-CoV-2-infections) was determined. Factors associated with breakthrough infections were analyzed using COX proportional hazard models. Among 8,062,600 fully vaccinated adults, we identified 373,070 breakthrough infections with an incidence of 11.2 (95%CI 11.2–11.3)/100 person years. Vaccination with Ad26.COV2.S (HR1.54, 95%CI 1.52–1.56) or ChAdOx1 (HR1.68, 95%CI 1.66–1.69) was associated with a higher risk of a breakthrough infection compared to BNT162b2, while mRNA-1273 was associated with a lower risk (HR0.68, 95%CI 0.67–0.69). A prior COVID-19-infection was protective against a breakthrough infection (HR0.23, 95%CI 0.23–0.24), as was an mRNA booster (HR0.44, 95%CI 0.43–0.45). During a breakthrough infection, those who had a prior COVID-19 infection were less likely to have COVID-19 symptoms of almost all types than naïve persons. We identified risk factors associated with breakthrough infections, such as vaccination with adenoviral-vector vaccines, which could help inform future decisions on booster vaccination strategies. A prior COVID-19 infection lowered the risk of breakthrough infections and of having symptoms, highlighting the protective effect of hybrid immunity.

## 1. Introduction

Mass vaccination is being rolled out globally to mitigate the impact of the COVID-19 pandemic. In Belgium, vaccination was initiated on 28 December 2020 and by 11 August 2021, an 80% vaccination coverage for a complete primary scheme was achieved in the initially targeted adult population, aged 18 years and above. By 5 April 2022, vaccination coverage had risen to 89%. Two mRNA vaccines (two doses: BNT162b2 and mRNA-1273) and two viral vector vaccines (ChAdOx1 (two doses) and Ad26.COV2.S (one dose)) were used. For these vaccines, clinical trials have shown high vaccine efficacy against symptomatic infection [1,2,3,4] and high vaccine effectiveness has been demonstrated under real-world conditions [5,6].

However, no vaccine can prevent infection entirely and breakthrough infections still occur. Since the beginning of the vaccination campaign, Belgium has experienced a moderate third wave of COVID-19 infections starting mid-February 2021, and a large fourth wave that started at the beginning of October 2021, despite the high national vaccination coverage of more than 80%. Emerging new variants of concern with increased transmissibility and immune escape, as well as a partial lift in social restriction measurements, waning of vaccine effectiveness and seasonal effects, likely contributed to these surges in post-vaccination infections [7]. Therefore, booster doses were recommended in Belgium since 22 September 2021, initially for groups at higher risk of (severe) infection (nursing home residents, persons >65 years and healthcare workers) and were then rapidly extended to the entire adult population from 27 November 2021.

Until now, there were limited data on the risk factors of a COVID-19 breakthrough infection after full vaccination, and most studies were based on small cohorts that were only followed up during the first half of 2021 [8,9,10,11,12,13]. Insight into such risk factors could guide public health policies regarding vaccination booster strategies especially related to the identification of the target population, the period and frequency.

The objective of this study was to investigate the incidence and risk factors associated with breakthrough infections, and to assess the symptomatic profile of individuals with breakthrough infections, using the nationwide epidemiological Belgian surveillance system. Potential risk factors included demographical and clinical characteristics, brand of administered vaccines, a person’s exposure to the SARS-CoV-2 virus through their profession or environment and a prior COVID-19 infection before vaccination.

## 2. Methods

### 2.1. Study Design and Population

We used data from the LINK-VACC project: a prospective, real-time cohort with national-level coverage in Belgium. The population of interest comprised all individuals aged 18 years and above, living in Belgium, who had been fully vaccinated with a primary vaccination scheme for at least 14 days with a COVID-19 vaccine approved by the European Medicine Agency, between 1 February 2021 and 5 December 2021. We chose a 14-day interval to allow time for an immune response to develop. Two mRNA vaccines (BNT162b2: 2 doses of 30 µg/dose and mRNA-1273: 2 doses of 100 µg/dose) and two viral vector vaccines (ChAdOx1: 2 doses of >2.5 × 10^8^ infectious units and Ad26.COV2.S: 1 dose of >8.92 log_10_ infectious units) were used.

### 2.2. Data Sources

The LINK-VACC project was launched by Sciensano and its collaborators to perform the post-authorization surveillance of COVID-19 vaccines in Belgium. To that aim, selected variables from multiple existing national health and social sector registries were linked at an individual level using the unique Belgian social security number [14]. The databases were linked within a pseudonymized environment hosted by Healthdata.be of Sciensano. For this study, the databases Vaccinnet+, COVID-19 Healthdata and the Belgian Common Base Registry for HealthCare Actor (CoBRHA) were used. Vaccinnet+ is the national COVID-19 vaccination registry, containing all COVID-19 vaccination records registered in Belgium, with information on demographics of vaccinated persons, the vaccine brand and date of administered vaccine doses. In the COVID-19 HealthData databases, data on COVID-19 laboratory tests (date of sampling and test result) and on contact tracing were recorded. Contact tracing data included information on presence and type of symptoms during COVID-19 infection at the moment of the telephone call by national contact tracing (on average within 24 h after a positive test), collected using a predefined list of symptoms consistent with COVID-19 [15]. The CoBRHA database allowed the identification of people who have been licensed to practice a healthcare profession in Belgium.

### 2.3. Outcomes

Breakthrough infections were defined as laboratory (PCR or rapid antigen test) confirmed SARS-CoV-2 infections occurring after being fully vaccinated for at least 14 days. If there was another positive test within the 90 days prior to a breakthrough infection, this breakthrough infection was not included in the analyses, to avoid identification of a prior infection acquired before vaccination.

Symptomatic breakthrough infections were defined as laboratory confirmed breakthrough infections in the presence of clinical symptoms, based on the Belgian case-definition criteria of a possible COVID-19 case: at least one major symptom (cough, dyspnea, anosmia/dysgeusia) or at least two minor symptoms (fever, muscle or joint pain, rhinitis, sore throat, headache or diarrhea) [16]. The symptomatic profile of persons with a breakthrough infection was explored using the reported symptom distribution.

### 2.4. Risk Factors

We collected persons’ characteristics of interest including age at first vaccination, sex as recorded in in the population registry, healthcare worker status, brand of primary vaccine scheme and whether they had received a booster vaccine. History of a prior COVID-19 infection before vaccination was defined based on presence of a positive test prior to the date of the first dose of the primary vaccine scheme. The total number of SARS-CoV-2 tests (negative or positive) performed during the study period for each person was used as a proxy for a person’s frequency testing profile, categorized as high when having ≥4 tests (e.g., persons subject to routine COVID-19 screening in the context of high-risk occupational exposure or as a prevention policy in medical or residential care facilities). To estimate the background level of circulating virus for a specific person, we calculated the positivity rate (0–100) for COVID-19 cases within this person’s geographical region of residence (Flanders/Brussels/Wallonia) and age group, for the entire follow up of this person during the study.

### 2.5. Statistical Analysis

Demographic and clinical characteristics were summarized for fully vaccinated persons and for persons with a (symptomatic) breakthrough infection using number of individuals and proportions. We determined the breakthrough infection incidence per 100 person years, based on a person’s follow-up time, from ≥14 days after the last primary vaccine until occurrence of a first breakthrough infection, or otherwise until death or the end of follow up on 5 December 2021. Incidence rates were assessed for the entire study period and stratified by periods with a different variant of concern (VOC) dominance: 1 February 2021 to 18 June 2021 for the alpha variant and 15 July 2021 to 5 December 2021 for the delta variant [17]. The study period ended before the omicron variant became dominant.

Cox proportional hazard models were conducted to derive cumulative hazard plots and to derivate adjusted hazard ratios (HRs) with 95% confidence intervals (95%CI) for potential risk factors associated with the development of a first (symptomatic) breakthrough infection. Cox proportional hazard assumptions were checked. A sensitivity analysis was performed by censuring at time of booster administration in order to analyze the risk factors of a breakthrough infection after a primary vaccine scheme without booster vaccines. Another sensitivity analysis was performed among boosted persons only.

When analyzing the incidence of a symptomatic breakthrough infection, in case no information on symptoms was collected, the breakthrough infection was considered as a non-symptomatic breakthrough infection (non-event). We assessed among breakthrough infections with available information on symptoms, the likelihood of having a specific symptom or having ≥1 symptom (versus no symptoms), between persons with and without a prior COVID-19 infection using logistic regression models adjusted for age, sex and vaccine brand. These models were subsequently stratified by age group (18–64, 65–79 and ≥80 years) given the association of older age with the occurrence of more COVID-19 symptoms, which could confound observed associations.

Significance level was set at 0.05, based on two-sided tests. Analyses were carried out using R version 4.1.2 (R Foundation for Statistical Computing, Vienna, Austria. https://www.R-project.org/ (accessed on 10 March 2022)).

## 3. Results

### 3.1. Population Characteristics

**Figure 1:**
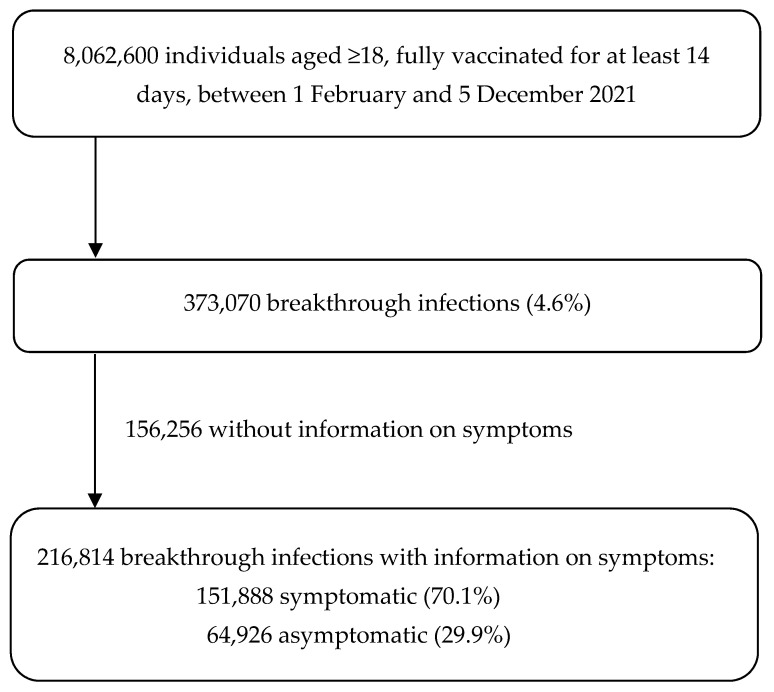
Flow chart of individuals fully vaccinated for at least 14 days on 5 December 2021 in Belgium, with the occurrence of (symptomatic) breakthrough COVID-19 infections. Breakthrough infection = laboratory confirmed COVID-19 infection after at least 14 days after the last vaccine dose; symptomatic breakthrough infection = laboratory confirmed COVID-19 infection after at least 14 days after the last vaccine dose with compatible COVID-19 symptoms.

We included 8,062,600 persons that were fully vaccinated between 1 February 2021 and 5 December 2021 and aged 18 years and above, which represented 87.6% of the total Belgian 18 year plus adult population [18] (Figure 1). Included persons were followed up for a total of 3,320,273 person years (Table 1). The mean follow-up time was 150 days (±49) and the maximum was 306 days.

Demographic and clinical characteristics of fully vaccinated persons are summarized in Table 1. The majority of included persons were fully vaccinated with BNT162b2 (69.1%), followed by ChAdOx1 (17.4%), mRNA-1273 (8.3%) and Ad26.COV2.S (5.1%). Persons vaccinated with mRNA-based primary vaccine schemes were followed up for slightly longer than persons vaccinated initially with viral-vector-based vaccines since the latter were introduced later during the Belgian vaccination campaign (mean follow up of 154 (±53) days versus 139 (±32) days, respectively). Of all fully vaccinated individuals, 11.9% had received an mRNA-based booster vaccine at least 14 days before the end of follow up, after a median time of 179 (IQR 63) days after the last dose of a primary scheme.

### 3.2. Occurrence of Breakthrough Infections and Persons’ Characteristics

Of all fully vaccinated persons, 373,070 (4.6%) developed a breakthrough infection during follow up (Figure 1). Overall, the incidence of breakthrough infections was 11.2 per 100 person years (95%CI 11.2–11.3) (Table 1). The median time to infection was 121 days (IQR 97–156). We observed a comparable incidence by sex, and higher incidences for younger age groups than for older age groups (18.4 versus 4.6 per 100 person years, for age groups 35–44 and ≥85, respectively). The incidence of breakthrough infections was higher within individuals vaccinated with viral-vector-based vaccines (12.7 and 16.5 per 100 person years for ChAdOx1 and Ad26.COV2.S, respectively), compared to those vaccinated with mRNA-based vaccines (11.0 and 7.6 per 100 person years for BNT162b2 and mRNA-1273, respectively). Among persons who had a prior COVID-19 infection before vaccination, a lower incidence of breakthrough infections was observed compared to COVID-19 naïve persons (3.2 versus 12.0 per 100 person years).

**Figure 2:**
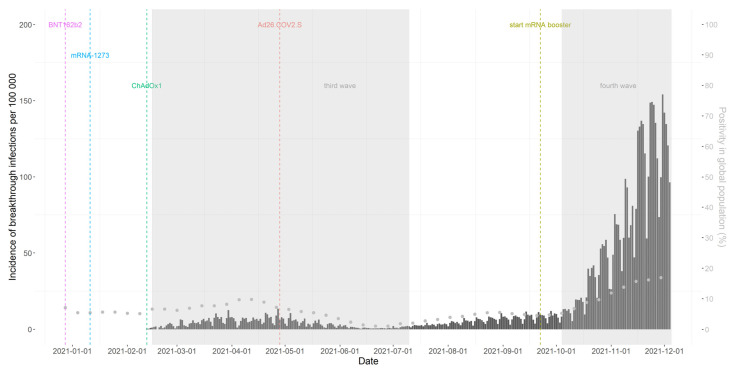
Daily incidence of breakthrough infections per 100,000 persons during 2021 among fully vaccinated persons (bars) and the weekly positivity rate in the global population (dots). Dashed lines represent the introduction of different primary vaccine brands or of the mRNA booster. Shaded areas represent the occurrence of waves of COVID-19 infections in Belgium.

When analyzing the daily incidence of breakthrough infection by calendar date, the highest incidences were observed during the fourth COVID-19 wave that started on 4 October 2021, coinciding with a high positivity rate in the general population (Figure 2 and Figure S1). The majority of breakthrough cases occurred 4 months after full vaccination (Figure S2). When comparing the incidence of breakthrough infections between different VOC dominance periods, a higher incidence was observed in the delta period than in the alpha period (14.2 versus 1.8 per 100 person years) (Tables S1 and S2).

### 3.3. Occurrence of Symptomatic Breakthrough Infections

Of the 373,070 persons with a breakthrough infection, 216,814 (58.1%) had available information on symptoms. Of these persons 70.1% (151,888/216,814) had symptoms compatible with COVID-19 (Figure 1), with an overall incidence rate of symptomatic breakthrough infections of 4.6 per 100 person years. Persons with a symptomatic breakthrough infection had a comparable distribution of demographic and clinical characteristics as all persons with a breakthrough infection and as persons with asymptomatic breakthrough infections (Table 1 and Table S3).

### 3.4. Risk Factors of a Breakthrough Infection

**Figure 3:**
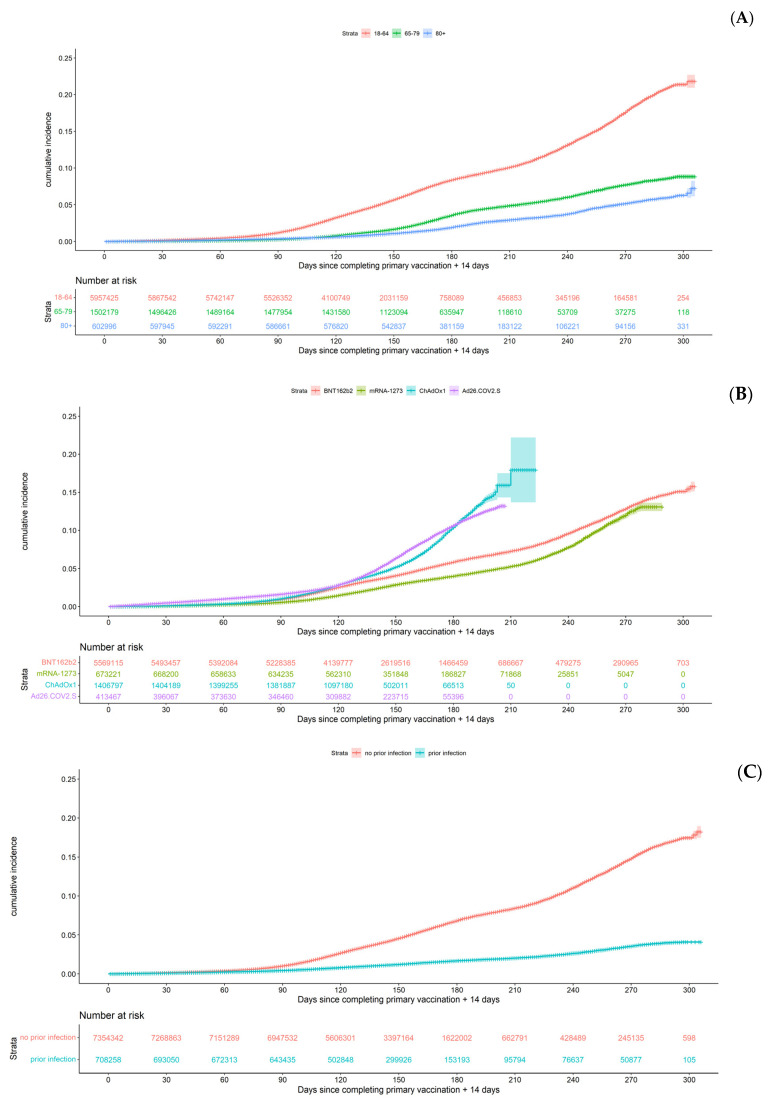
Cumulative incidence of breakthrough infections among fully vaccinated individuals (**A**) by different age groups, (**B**) by brand of primary vaccine and (**C**) by prior COVID-19 infection. Shaded areas represent 95% confidence intervals.

The unadjusted cumulative incidence of a breakthrough infection at 300 days after primary vaccination was 15.8% and for a reported symptomatic breakthrough infection it was 5.6%. The younger age group of 18–64 year olds had a higher cumulative incidence of a breakthrough infection than persons aged 65–79 or ≥80 years (Figure 3A). The cumulative incidences by brand of the primary vaccination scheme were higher for persons vaccinated with the Ad26.COV2.S vaccine or ChAdOx1 compared to persons vaccinated with BNT162b2 or mRNA-1273 (Figure 3B). Notably, persons with a prior COVID-19 infection had lower cumulative incidences of a breakthrough infection than naïve persons (Figure 3C). Comparable observations were made for symptomatic breakthrough infections (Figure S3).

Within the adjusted Cox proportional hazards model, younger age, females, non-healthcare workers, a higher background positivity rate and a high frequency testing profile were associated with a higher risk of a breakthrough infection (Table 2). When compared to BNT162b2, vaccination with Ad26.COV2.S (HR 1.54, 95%CI 1.52–1.56) or ChAdOx1 (HR 1.68, 95%CI 1.66–1.69) was associated with a higher risk of a breakthrough infection, while vaccination with mRNA-1273 was associated with a slightly lower risk (HR 0.68, 95%CI 0.67–0.69). A prior COVID-19 infection was protective against breakthrough infections (HR 0.23, 95%CI 0.23–0.24), as well as having received an mRNA booster vaccine (HR 0.44, 95%CI 0.43–0.45). The adjusted HRs for symptomatic breakthrough infections showed similar trends. The sensitivity analysis when censuring at administration of the booster also showed comparable results. Among only boosted persons, a protective effect of a prior COVID-19 infection and an increased risk for younger persons were also observed (Table S4).

### 3.5. Symptomatic Profile of Breakthrough Infections

**Figure 4:**
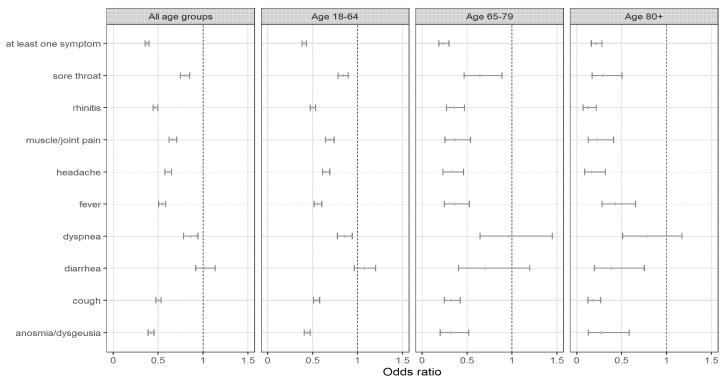
Symptoms in persons with a breakthrough infection and a prior COVID-19 infection versus persons with a breakthrough infection without a prior COVID-19 infection. Logistic regression models adjusted for age, sex and vaccine brands, and stratified by age group, to assess the association of each type of symptom or of having ≥1 symptom (versus no symptom) with prior COVID-19 infection status. The error bars represent 95% confidence intervals. Persons with a breakthrough infection and having available data on symptoms were taken into account for this analysis.

The most frequently reported symptom by persons with a breakthrough infection was rhinitis, followed by cough and headache (Figure S4). Among persons with a breakthrough infection, those who had a prior COVID-19 infection were less likely to have symptoms (OR 0.38 for ≥1 symptom versus no symptoms, 95%CI 0.36–0.40) than COVID-19 naïve persons (Figure 4). When looking at specific types of symptoms during a breakthrough infection, all of the symptoms, except for diarrhea, were less likely reported by previously infected persons. These lower odds of symptoms for persons with a prior COVID-19 infection were observed within every age group, except for dyspnea in the older age groups.

## 4. Discussion

Based on data of over eight million fully vaccinated adults, we observed an incidence of breakthrough infections of 11.2 per 100 person years, with 4.6% of all included persons testing positive during a mean follow up of 150 days. Factors associated with an increased risk of a breakthrough infection were younger age, female sex, non-healthcare workers, vaccination with adenoviral-vector-based vaccines, a higher background virus level and being tested frequently for COVID-19. Having had a prior COVID-19 infection before vaccination and having received a booster vaccine were associated with a lower risk of a breakthrough infection. Among those with a breakthrough infection, having had a prior COVID-19 infection also lowered the odds of experiencing symptoms.

The observed incidence of 11.2 per 100 person years in our study was relatively comparable to the incidence of 9.8 per 100 person years observed in a large cohort study from NHS England including over 15 million persons, based on data up until 1 November 2021 and with a comparable median follow-up time of 149 days [19]. The observed proportion of 4.6% breakthrough infections in our cohort by 5 December 2021 could not be directly compared to the proportions reported in other fully vaccinated cohorts with a shorter follow up and during the spring/summer months (range of 0.2% to 1.0%, data collection was generally from January up until September 2021) [8,9,10,12,13,20,21]. However, we observed previously comparable rates from February up until 8 August 2021 of 0.2%, and up until 31 October 2021 of 1.3% during our national surveillance in Belgium using the same methodology [7,22]. Comparing incidence rates directly between cohorts, however, remains challenging because of study-specific differences (size and characteristics of the studied population, methods of data collection, follow-up time) and country-specific differences (roll-out of vaccination campaigns and vaccines used, seasonal effects, applied protective measures, vaccination coverage).

Having had a prior COVID-19 infection before vaccination reduced the risk of a breakthrough infection and possibly, inherently, also the risk of more severe outcomes. These results added to the growing evidence that naturally acquired immunity together with vaccine-induced immunity (hybrid immunity) has an additional protective effect, compared to only one of the two [10,23,24,25]. Among persons with breakthrough infections, those who had a prior COVID-19 infection were less likely to have symptoms than COVID-19 naïve persons, and this was the case for almost every type of symptom. We are, to the best of our knowledge, the first to demonstrate that hybrid immunity reduces the risk of nearly all types of COVID-19-related symptoms, both mild and severe.

Vaccination with adenoviral-vector-based vaccines was associated with a higher risk of breakthrough infections, compared to vaccination with mRNA-based vaccines, while adjusting for potential confounders. This result was in line with real-world data on the effectiveness of viral-vector-based vaccines (ChAdOx1, or Ad26.COV2.S) versus mRNA-based vaccines against the delta variant [17,26,27,28,29], and was also seen in other analyses of breakthrough infections [8,11,12,30,31]. This observation should be treated with caution, since this observational study was not designed as a formal comparison, and given the fact that vaccine brands were rolled-out at different time-points and administered to different demographic profiles, although we adjusted for such factors in the multivariate analyses. However, this finding contributed to the existing evidence on differences in protection offered between vaccine brands, and might help governments in developing or redirecting vaccination strategies.

Being younger was associated with an increased risk of a breakthrough infection, also in the multivariate analysis. A higher rate of breakthrough infections in younger adults has also been observed in other cohorts [8,11,12]. This increased risk might reflect different social behavior, such as more contacts with unvaccinated minors, more social contacts and more work-related contacts in younger age groups. This hypothesis was supported by data from the Belgian national contact tracing during this study period, indicating that 41% of all reported high-risk contacts occurred between index cases of 18–65 years old and their high-risk contacts of the same age group [15].

Having received a booster vaccine protected against developing a breakthrough infection, also in the multivariate model, which adjusted for age and healthcare worker status. However, this study was not designed to investigate the effectiveness of a booster vaccine against infection compared to a primary vaccine schedule. Additionally, the interpretation of this booster effect was hampered by the fact that booster vaccines were only administered after a median of 6 months of follow up. The sensitivity analysis of risk factors when censuring at booster administration showed similar findings as the main analysis, and in the subpopulation of boosted persons, a protective effect of a prior COVID-19 infection and an increased risk of breakthrough infections for younger persons were also seen, supporting our interpretation.

In the multivariate analysis, healthcare workers were associated with a lower risk of a breakthrough infection, despite being, on average, more exposed due to occupational contact with COVID-19 patients. This might reflect a good shielding in the healthcare workers group, with a higher vaccine coverage than in the general population and intensive use of protective materials while at work. A prospective cohort study among almost 6000 healthcare workers in Denmark showed that the numbers infected due to close contact with COVID-19 patients were comparable to the numbers infected following COVID-19 contact outside work [32].

Male sex was associated with a lower risk, although the effect size was much smaller than for the other tested factors (0.99; 95%CI (0.98–0.99)), which does not suggest a clearly pronounced effect of sex at the population level.

With respect to the observation that most breakthrough infections occurred after 4 months after full vaccination, it was important to notice that many persons in our cohort were fully vaccinated in July 2021, and that a fourth wave of COVID-19 infections occurred in October 2021. Therefore, this observation was probably not only attributable to waning vaccine effectiveness, but also to the delta VOC, different social restriction measurements and seasonal effects. When comparing the incidence rates between the two different VOC dominance periods, a higher incidence rate was seen during the delta period than during the alpha period. This added evidence to a previous study showing that the delta VOC (compared to the alpha VOC) was associated with decreased vaccine effectiveness against transmission of infection [17].

Only few smaller cohorts have reported on the proportion of symptomatic cases among breakthrough infections, ranging from 74% to 95% [9,10,21]. One cohort with data on 906 breakthrough infections after full vaccination also reported rhinitis, headache and cough among the 7 most commonly reported symptoms [9]. However, these cohorts differed in size and data collection methods, which hampered comparisons with our observations.

We chose to include only individuals over 18 years of age in the present analysis because they corresponded to the population initially targeted by the Belgian vaccination campaign. Vaccination of children and adolescents was made possible at a much later stage (June 2021) and limited to mRNA vaccines with an adapted dosage. Lower vaccination coverage among this specific population and potentially different immunogenicity and clinical responses would require a separate study. In addition, we chose to include data up until 5 December 2021, because several changes were implemented in the testing strategy after that date. For example, high-risk contacts were tested only once or, if they were symptom-free, not at all. This could have impacted estimates of the incidence of (symptomatic) breakthrough infections.

Our study had several strengths, including a large nationwide population covering 88% of the adult Belgian population, it was diverse in demographical characteristics and compared four EMA-approved vaccine brands, which added to the generalizability of our results. We used systematically collected, person-level data over a period of 10 months within the LINK-VACC project. This platform links data from several national registries, including the vaccination status as registered by the vaccinator, all systematically performed laboratory tests, official healthcare worker status and presence of symptoms from the national contact tracing. When investigating the risk of breakthrough infections we took time since vaccination, demographic and clinical characteristics of vaccinated persons and background level of virus circulation into account in order to adjust for these possible confounding factors.

However, it was possible that there was an underestimation of breakthrough infections identified in this study, because of variation in willingness to test between individuals and because of changing testing policies over time. Persons without symptoms were less likely to get tested, which could have led to an underestimation of the proportion of asymptomatic breakthrough infections. Previously infected persons could either be less likely to get tested because of self-perceived lower risk of re-infection, or more likely as they would recognize a COVID-19 infection more easily. Nonetheless, the mean number of performed tests per individual during the study was similar between those previously infected or not (respectively 0.48 ± 1.04 and 0.45 ± 0.97). We additionally adjusted for a frequent testing profile in the multivariate analyses.

Information on the presence of symptoms was not exhaustive for all detected breakthrough infections and was only collected at the time of the call by contact tracing, within approximately 24 h after a positive test, which might have impacted the estimated proportion and incidence rate of symptomatic breakthrough infections. However, information was available for 58% of all breakthrough infections and systematically collected via a predefined questionnaire by contact tracing. Moreover, breakthrough infections missing information on symptoms had similar demographic and clinical characteristics as breakthrough infections with available information on symptoms.

We were unable to report on the continuum from infection to more severe outcomes, such as hospitalizations or death, as these data were collected in an aggregated daily survey, which was not linkable at the individual level. However, the characterization of a cohort of all vaccinated persons who had to be hospitalized or who died because of COVID-19 at the hospital, will be subject to a subsequent study. We were also unable to take into account adverse determinants of health, which might impact the risk of breakthrough infections, including comorbidities, lifestyle or sociodemographic factors, as we were limited to the variables collected in pre-existing databases. Possible seasonal effects were not taken into account, although background level of virus circulation was considered.

Within the Belgian vaccination campaign it was decided to use primarily mRNA vaccines as booster vaccines, based on international effectiveness data and our national surveillance data. Identifying individuals at increased risk of infection after vaccination might help inform public health policy decisions regarding future booster vaccination strategies, both in Belgium and internationally. We plan to continue monitoring the Belgian vaccination campaign and to study the severity and multiple recurrences of breakthrough infections, as well as the role of emerging variants.

## 5. Conclusions

Our results from this nationwide Belgian study of fully vaccinated persons showed an incidence rate of breakthrough infections of 11.2 per 100 persons per year. The risk of a breakthrough infection was increased in younger individuals and for those vaccinated with adenoviral vector vaccines, after adjustment for exposure to the SARS-CoV-2 virus through their profession or environment. The risk of a breakthrough infection was significantly reduced for those having been previously infected, and for those having received a booster vaccine. Compared with COVID-19 naïve persons, persons having been previously infected and who were re-infected after vaccination were more likely to be completely asymptomatic. In addition, almost all types of symptoms were less common in the latter group. These findings highlighted the protective effect of hybrid immunity and could help inform future decisions on booster vaccination strategies.

## Figures and Tables

**Table 1:** Demographic and clinical characteristics of adult individuals who had been fully vaccinated and those with a breakthrough infection after full vaccination, as of 5 December 2021.

	Fully Vaccinated ^1^ (>14 Days)	Breakthrough Infection, *n* (%)	Incidence per 100 Person Years (95%CI)	Symptomatic Breakthrough Infection, *n* (%)	Incidence per 100 Person Years (95%CI)
Overall	8,062,600	373,070	11.2 (11.2–11.3)	151,888	4.6 (4.6–4.6)
Sex					
Female	4,139,926 (51.3%)	199,900 (53.6%)	11.3 (11.3–11.4)	86,178 (56.7%)	4.9 (4.8–4.9)
Male	3,922,674 (48.7%)	173,170 (46.4%)	11.2 (11.1–11.2)	65,710 (43.3%)	4.2 (4.2–4.3)
Age categories (years)					
18–24	742,418 (9.2%)	31,133 (8.3%)	12.9 (12.8–13.1)	11,803 (7.8%)	4.9 (4.8–5.0)
25–34	1,178,450 (14.6%)	63,119 (16.9%)	15.3 (15.1–15.4)	26,796 (17.6%)	6.5 (6.4–6.6)
35–44	1,256,702 (15.6%)	86,505 (23.2%)	18.4 (18.3–18.5)	39,680 (26.1%)	8.4 (8.4–8.5)
45–54	1,361,440 (16.9%)	71,714 (19.2%)	13.0 (12.9–13.1)	31,014 (20.4%)	5.6 (5.6–5.7)
55–64	1,418,415 (17.6%)	57,292 (15.4%)	9.4 (9.3–9.5)	22,955 (15.1%)	3.8 (3.7–3.8)
65–74	1,124,756 (14.0%)	35,622 (9.5%)	6.9 (6.8–6.9)	12,826 (8.4%)	2.5 (2.4–2.5)
75–84	671,571 (8.3%)	19,761 (5.3%)	5.7 (5.6–5.8)	5651 (3.7%)	1.6 (1.6–1.7)
≥85	308,848 (3.8%)	7924 (2.1%)	4.6 (4.5–4.7)	1163 (0.8%)	0.7 (0.6–0.7)
Brand of primary vaccine					
BNT162b2	5,569,115 (69.1%)	257,119 (68.9%)	11.0 (10.9–11.0)	102,860 (67.7%)	4.4 (4.4–4.4)
mRNA-1273	673,221 (8.3%)	21,614 (5.8%)	7.6 (7.5–7.7)	8866 (5.8%)	3.1 (3.0–3.2)
ChAdOx1	1,406,797 (17.4%)	68,525 (18.4%)	12.7 (12.7–12.8)	29,390 (19.3%)	5.5 (5.4–5.5)
Ad26.COV2.S	413,467 (5.1%)	25,812 (6.9%)	16.5 (16.3–16.7)	10,772 (7.1%)	6.9 (6.8–7.0)
Healthcare worker					
No	7,577,369 (94.0%)	339,041 (90.9%)	11.1 (11.1–11.2)	136,183 (89.7%)	4.5 (4.4–4.5)
Yes	485,231 (6.0%)	34,029 (9.1%)	12.4 (12.2–12.5)	15,705 (10.3%)	5.7 (5.6–5.8)
Prior COVID-19 infection ^2^					
No	7,354,342 (91.2%)	363,710 (97.5%)	12.0 (12.0–12.0)	149,259 (98.3%)	4.9 (4.9–5.0)
Yes	708,258 (8.8%)	9360 (2.5%)	3.2 (3.2–3.3)	2629 (1.7%)	0.9 (0.9–0.9)
Number of COVID-19 tests ^3^					
0–3	7,928,881 (98.3%)	345,544 (92.6%)	10.6 (10.6–10.6)	142,756 (94.0%)	4.4 (4.4–4.4)
≥4	133,719 (1.7%)	27,526 (7.4%)	45.0 (44.5–45.5)	9132 (6.0%)	14.9 (14.6–15.2)
Booster vaccine received					
No	7,104,588 (88.1%)	353,608 (94.8%)	12.7 (12.6–12.7)	146,431 (96.4%)	5.2 (5.2–5.3)
Yes	958,012 (11.9%)	19,462 (5.2%)	3.7 (3.6–3.7)	5457 (3.6%)	1.0 (1.0–1.1)

^1^ Fully vaccinated = vaccinated with a complete primary vaccination scheme for at least 14 days with a COVID-19 vaccine approved by the European Medicine Agency, between 1 February 2021 and 5 December 2021. ^2^ Prior COVID-19 infection = positive PCR or Rapid Antigen test recorded in the COVID-19 laboratory test result database prior to the date of the first dose of a primary vaccine schedule. Serology and auto-tests were not considered. ^3^ Number of COVID-19 tests = the total number of SARS-CoV-2 tests (negative or positive) performed during the study period per person, which was used as a proxy for a person’s high or a low frequency testing profile (e.g., persons subject to routine COVID-19 screening in the context of high-risk occupational exposure or as a prevention policy in medical or residential care facilities), with a cut-off of ≥4 tests.

**Table 2:** Factors associated with breakthrough infection (multivariable Cox proportional hazards regression).

Factor	Hazard Ratio (95%CI)	*p*-Value
Age (per 10-year increase)	0.88 (0.88–0.88)	<0.001
Male sex	0.99 (0.98–0.99)	<0.001
Brand primary vaccine (ref: BNT162b2)		
mRNA-1273	0.68 (0.67–0.69)	<0.001
ChAdOx1	1.68 (1.66–1.69)	<0.001
Ad26.COV2.S	1.54 (1.52–1.56)	<0.001
Healthcare worker	0.60 (0.60–0.61)	<0.001
Prior COVID-19 infection	0.23 (0.23–0.24)	<0.001
Received mRNA booster	0.44 (0.43–0.45)	<0.001
Background positivity rate	1.33 (1.32–1.33)	<0.001
High frequency testing profile	3.87 (3.82–3.92)	<0.001

## Data Availability

As the LINK-VACC project is not an open-access platform, the individual level data are only available to researchers working on the project. However, general descriptive statistics from the registries used in LINK-VACC are available from https://epistat.wiv-isp.be/covid/. This includes, among others, the number of confirmed cases by date, age, sex and province or the number of administered vaccines by date, region, age, sex, brand and dose.

## Supplementary Materials

The following supporting information can be downloaded at: https://www.mdpi.com/article/10.3390/v14040802/s1, Figure S1: Daily incidence of symptomatic or asymptomatic breakthrough infections per 100,000 persons during 2021 among fully vaccinated persons; Figure S2: Number of breakthrough infections among fully vaccinated persons by month after 14 days after last dose of the primary vaccine scheme; Table S1: Demographic and clinical characteristics of adult individuals who have been fully vaccinated and those with a breakthrough infection during alpha VOC dominance period; Table S2: Demographic and clinical characteristics of adult individuals who have been fully vaccinated and those with a breakthrough infection, during delta VOC dominance period; Table S3: Demographic and clinical characteristics of adult individuals with a symptomatic or an asymptomatic breakthrough infection after full vaccination; Figure S3: Cumulative incidence of symptomatic breakthrough infections among fully vaccinated individuals; Table S4: Sensitivity analyses based on multivariable Cox proportional hazards regressions; Figure S4: Percentages and counts of each type of symptom or of having no symptoms among persons with a breakthrough infection, grouped by having had a prior COVID-19 infection before vaccination (reinfected) or not (naïve).
