# Supplementary material for: Incidence and Risk Factors of COVID-19 Vaccine Breakthrough Infections: A Prospective Cohort Study in Belgium
Source: Viruses. 2022 Apr 13;14(4):802. doi: 10.3390/v14040802 (PMC9029338; doi:10.3390/v14040802)
Supplement: Supplementary file 1 [file viruses-14-00802-s001.zip › viruses-1653187-supplementary.pdf]

Supplementary Materials

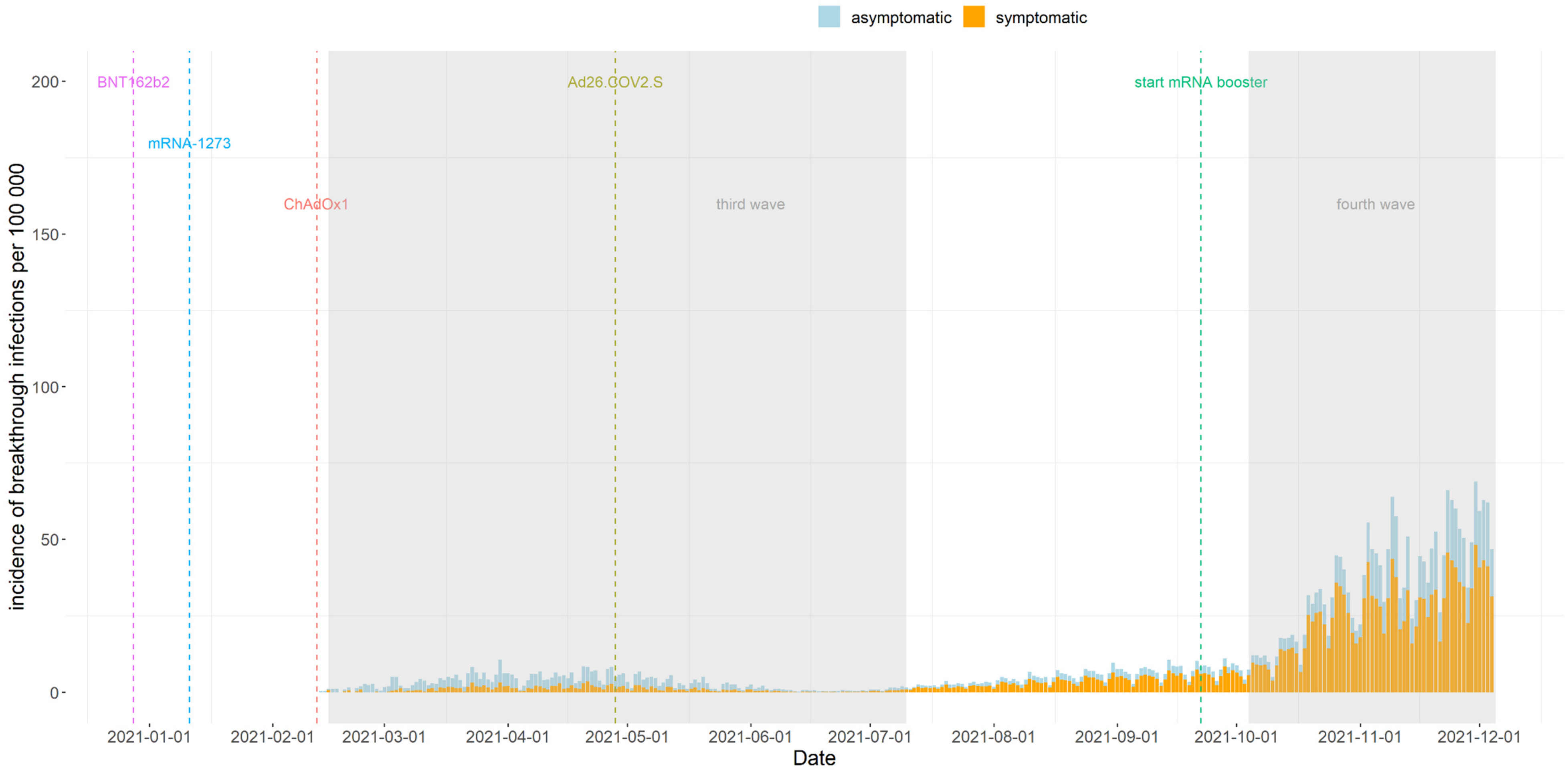

Figure S1: Daily incidence of symptomatic or asymptomatic breakthrough infections per 100,000 persons during 2021 among fully vaccinated persons (bars). Dashed lines represent introduction of different primary vaccine brands or of mRNA booster. Shaded areas represent occurrence of waves of COVID-19 infections in Belgium.

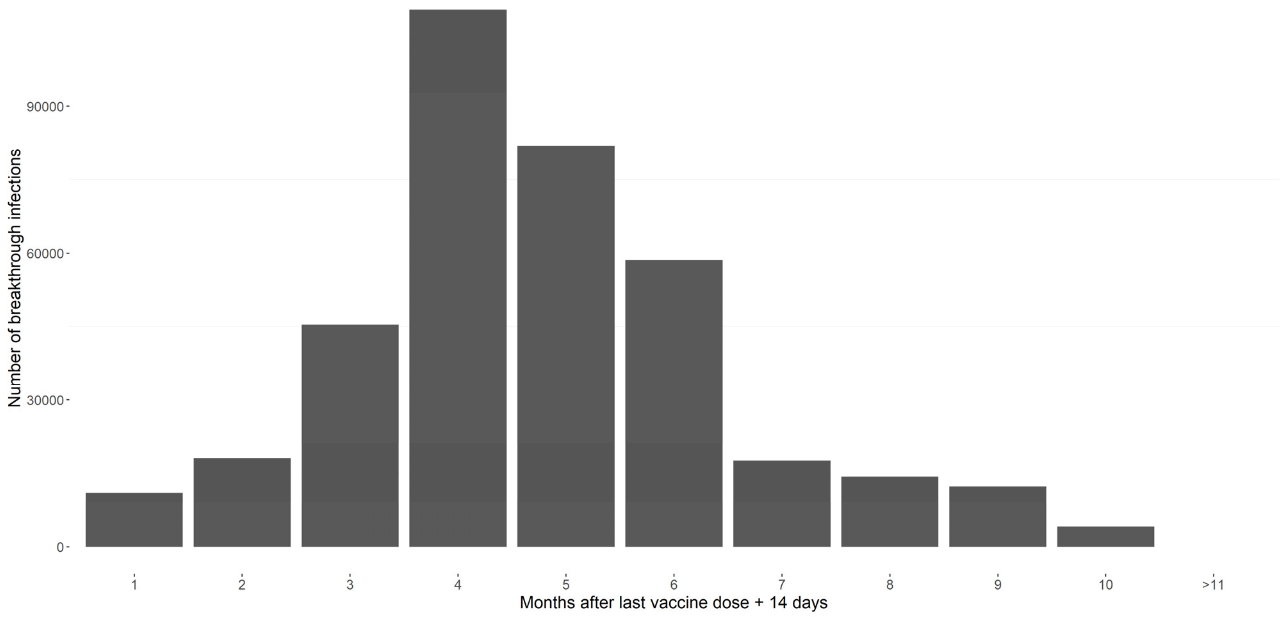

Figure S2: Number of breakthrough infections among fully vaccinated persons by month after 14 days after last dose of the primary vaccine scheme.

Table S1: Demographic and clinical characteristics of adult individuals who have been fully vaccinated and those with a breakthrough infection during alpha VOC dominance period, between February, 1, 2021 and June, 18, 2021.

	**Fully vaccinated**[1] **(> 14 days)**	**Breakthrough infection, n (%)**	**Incidence per 100 person-years (95%CI)**
**overall**	2,455,751	4,654	1.8 (1.8-1.9)
Sex			
Female	1,452,346 (59.1%)	3,278 (70.4%)	1.9 (1.9-2.0)
Male	1,003,405 (40.9%)	1,376 (29.6%)	1.6 (1.5-1.7)
Age categories (years)			
18-24	76,543 (3.1%)	235 (5.0%)	2.2 (1.9-2.5)
25-34	168,335 (6.9%)	555 (11.9%)	2.2 (2.0-2.4)
35-44	193,650 (7.9%)	631 (13.6%)	2.4 (2.2-2.6)
45-54	283,981 (11.6%)	608 (13.1%)	1.9 (1.8-2.1)
55-64	403,765 (16.4%)	535 (11.5%)	1.4 (1.3-1.5)
65-74	619,217 (25.2%)	388 (8.3%)	1.0 (0.9-1.1)
75-84	514,598 (21.0%)	633 (13.6%)	1.3 (1.2-1.4)
≥85	195,662 (8.0%)	1,069 (23.0%)	2.9 (2.7-3.0)
Brand of primary vaccine			
BNT162b2	1,967,424 (80.1%)	4,336 (93.2%)	1.9 (1.9-2.0)
mRNA-1273	206,312 (8.4%)	214 (4.6%)	1.0 (0.9-1.1)
ChAdOx1	188,659 (7.7%)	40 (0.9%)	0.9 (0.6-1.1)
Ad26.COV2.S	93,356 (3.8%)	64 (1.4%)	1.6 (1.2-2.0)
Healthcare worker			
No	2,093,262 (85.2%)	3,276 (70.4%)	1.7 (1.7-1.8)
Yes	362,489 (14.8%)	1,378 (29.6%)	2.1 (2.0-2.2)
Prior COVID-19 infection[2]			
No	2,256,293 (91.9%)	3,865 (83.0%)	1.7 (1.7-1.8)
Yes	199,458 (8.1%)	789 (17.0%)	2.7 (2.5-2.9)
Number of COVID-19 tests[3]			
0-3	2,392,818 (97.4%)	2,788 (59.9%)	1.1 (1.1-1.2)
≥4	62,933 (2.6%)	1,866 (40.1%)	18.5 (17.7-19.4)

[1] Fully vaccinated = vaccinated with a complete primary vaccination scheme for at least 14 days with a COVID-19 vaccine approved by the European Medicine Agency, between February 1, 2021 and December 5, 2021.
[2] Prior COVID-19 infection = positive PCR or Rapid Antigen test recorded in the COVID-19 Laboratory test result database prior to date of first dose of primary vaccine schedule. Serology and auto-tests are not considered.
[3] Number of COVID-19 tests = The total number of SARS-CoV-2 tests (negative or positive) performed during the study period per person was used as a proxy for a person's high or a low frequency testing profile(e.g. persons subject to routine COVID-19 screening in the context of high-risk occupational exposure or as a prevention policy in medical or residential care facilities), with a cut-off of ≥4 tests.

Table S2: Demographic and clinical characteristics of adult individuals who have been fully vaccinated and those with a breakthrough infection, during delta VOC dominance period, between July, 15, 2021 and December, 5, 2021.

	**Fully vaccinated[1] (> 14 days)**	**Breakthrough infection, n (%)**	**Incidence per 100 person-years (95%CI)**
**overall**	3,943,920	174,996	14.2 (14.2-14.3)
Sex			
Female	1,840,575 (46.7%)	86,168 (49.2%)	15.0 (14.9-15.1)
Male	2,103,345 (53.3%)	88,828 (50.8%)	13.5 (13.4-13.6)
Age categories (years)			
18-24	596,657 (15.1%)	19,093 (10.9%)	11.5 (11.3-11.6)
25-34	910,409 (23.1%)	40,892 (23.4%)	15.2 (15.0-15.3)
35-44	864,376 (21.9%)	52,211 (29.8%)	19.2 (19.0-19.4)
45-54	683,151 (17.3%)	31,842 (18.2%)	14.4 (14.3-14.6)
55-64	562,095 (14.3%)	21,215 (12.1%)	11.2 (11.1 11.4)
65-74	274,223 (7.0%)	8,514 (4.9%)	9.0 (8.8-9.1)
75-84	38,501 (1.0%)	881 (0.5%)	7.2 (6.7-7.6)
≥85	14,508 (0.4%)	348 (0.2%)	8.2 (7.3-9.0)
Brand of primary vaccine			
BNT162b2	2,751,540 (69.8%)	125,180 (71.5%)	14.7 (14.7-14.8)
mRNA-1273	256,333 (6.5%)	7,578 (4.3%)	9.4 (9.2-9.6)
ChAdOx1	789,742 (20.0%)	36,361 (20.8%)	13.8 (13.6-13.9)
Ad26.COV2.S	146,305 (3.7%)	5,877 (3.4%)	16.3 (15.9-16.7)
Healthcare worker			
No	3,875,312 (98.3%)	171,854 (98.2%)	14.2 (14.1-14.3)
Yes	68,608 (1.7%)	3,142 (1.8%)	15.7 (15.1-16.2)
Prior COVID-19 infection[2]			
No	3,568,833 (90.5%)	171,271 (97.9%)	15.3 (15.3-15.4)
Yes	375,087 (9.5%)	3,725 (2.1%)	3.3 (3.2-3.4)
Number of COVID-19 tests[3]			
0-3	3,899,404 (98.9%)	167,994 (96.0%)	13.8 (13.8-13.9)
≥4	44,516 (1.1%)	7,002 (4.0%)	49.2 (48.0-50.3)

[1] Fully vaccinated = vaccinated with a complete primary vaccination scheme for at least 14 days with a COVID-19 vaccine approved by the European Medicine Agency, between February 1, 2021 and December 5, 2021.
[2] Prior COVID-19 infection = positive PCR or Rapid Antigen test recorded in the COVID-19 Laboratory test result database prior to date of first dose of primary vaccine schedule. Serology and auto-tests are not considered.
[3] Number of COVID-19 tests = The total number of SARS-CoV-2 tests (negative or positive) performed during the study period per person was used as a proxy for a person's high or a low frequency testing profile(e.g. persons subject to routine COVID-19 screening in the context of high-risk occupational exposure or as a prevention policy in medical or residential care facilities), with a cut-off of ≥4 tests.

Table S3: Demographic and clinical characteristics of adult individuals with a symptomatic or an asymptomatic breakthrough infection after full vaccination, as of December, 5, 2021.

	Breakthrough infection, n (%)	Breakthrough infection with information on symptoms, n (%)	Symptomatic breakthrough infection, n (%)	Asymptomatic breakthrough infection, n (%)
**overall**	373,070	216,814	151,888	64,926
Sex				
Female	199,900 (53.6%)	119,507 (55.1%)	86,178 (56.7%)	33,329 (51.3%)
Male	173,170 (46.4%)	97,307 (44.9%)	65,710 (43.3%)	31,597 (48.7%)
Age categories (years)				
18-24	31,133 (8.3%)	16,915 (7.8%)	11,803 (7.8%)	5,112 (7.9%)
25-34	63,119 (16.9%)	36,692 (16.9%)	26,796 (17.6%)	9,896 (15.2%)
35-44	86,505 (23.2%)	54,675 (25.2%)	39,680 (26.1%)	14,995 (23.1%)
45-54	71,714 (19.2%)	42,839 (19.8%)	31,014 (20.4%)	11,825 (18.2%)
55-64	57,292 (15.4%)	32,458 (15.0%)	22,955 (15.1%)	9,503 (14.6%)
65-74	35,622 (9.5%)	19,777 (9.1%)	12,826 (8.4%)	6,951 (10.7%)
75-84	19,761 (5.3%)	10,107 (4.7%)	5,651 (3.7%)	4,456 (6.9%)
≥85	7,924 (2.1%)	3,351 (1.5%)	1,163 (0.8%)	2,188 (3.4%)
Brand of primary vaccine				
BNT162b2	257,119 (68.9%)	148,414 (68.5%)	102,860 (67.7%)	45,554 (70.2%)
mRNA-1273	21,614 (5.8%)	13,256 (6.1%)	8,866 (5.8%)	4,390 (6.8%)
ChAdOx1	68,525 (18.4%)	40,334 (18.6%)	29,390 (19.3%)	10,944 (16.9%)
Ad26.COV2.S	25,812 (6.9%)	14,810 (6.8%)	10,772 (7.1%)	4,038 (6.2%)
Healthcare worker				
No	339,041 (90.9%)	195,626 (90.2%)	136,183 (89.7%)	59,443 (91.6%)
Yes	34,029 (9.1%)	21,188 (9.8%)	15,705 (10.3%)	5,483 (8.4%)
Prior COVID-19 infection[1]				
No	363,710 (97.5%)	211,456 (97.5%)	149,259 (98.3%)	62,197 (95.8%)
Yes	9,360 (2.5%)	5,358 (2.5%)	2,629 (1.7%)	2,729 (4.2%)
Number of COVID-19 tests[2]				
0-3	345,544 (92.6%)	201,266 (92.8%)	142,756 (94.0%)	58,510 (90.1%)
≥4	27,526 (7.4%)	15,548 (7.2%)	9,132 (6.0%)	6,416 (9.9%)
Booster vaccine received				
No	353,608 (94.8%)	206,251 (95.1%)	146,431 (96.4%)	59,820 (92.1%)
Yes	19,462 (5.2%)	10,563 (4.9%)	5,457 (3.6%)	5,106 (7.9%)

[1] Prior COVID-19 infection = positive PCR or Rapid Antigen test recorded in the COVID-19 Laboratory test result database prior to date of first dose of primary vaccine schedule. Serology and auto-tests are not considered.
[2] Number of COVID-19 tests = The total number of SARS-CoV-2 tests (negative or positive) performed during the study period per person was used as a proxy for a person's high or a low frequency testing profile(e.g. persons subject to routine COVID-19 screening in the context of high-risk occupational exposure or as a prevention policy in medical or residential care facilities), with a cut-off of ≥4 tests.

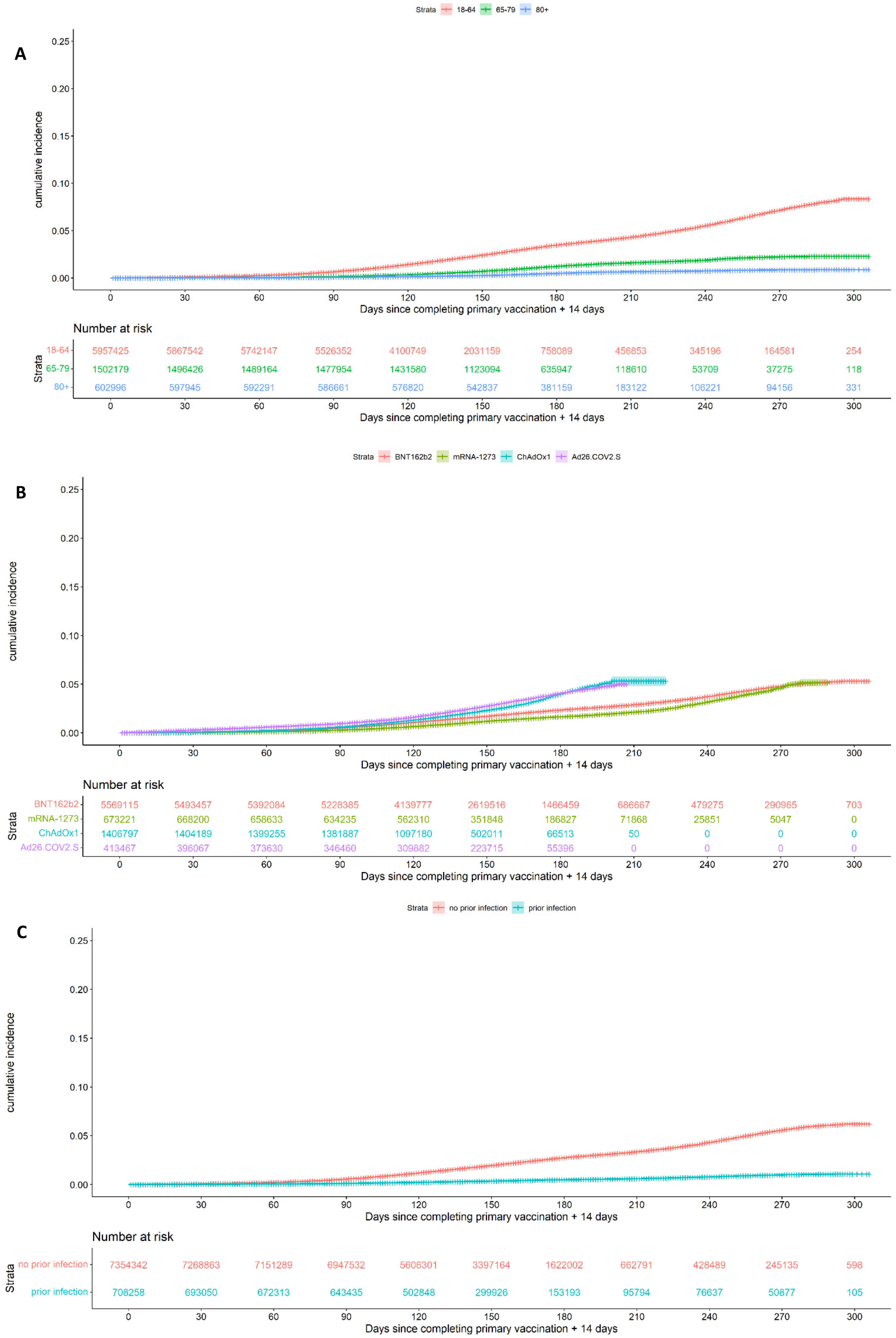

Figure S3: Cumulative incidence of symptomatic breakthrough infections among fully vaccinated individuals **(A)** by different age groups, **(B)** by brand of primary vaccine and **(C)** by prior COVID-19 infection. Shaded areas represent 95% confidence intervals.

Table S4: Sensitivity analyses based on multivariable Cox proportional hazards regressions.

A) factors associated with symptomatic breakthrough infection

Factor	Hazard ratio (95%)	p-value
Age (per 10 years increase)	0.85 (0.84-0.85)	<0.001
Male sex	0.86 (0.85-0.87)	<0.001
Brand primary vaccine (ref: BNT162b2)		
mRNA-1273	0.68 (0.67-0.70)	<0.001
ChAdOx1	1.77 (1.75-1.79)	<0.001
Ad26.COV2.S	1.56 (1.53-1.59)	<0.001
Healthcare worker	0.74 (0.72-0.75)	<0.001
Prior COVID-19 infection	0.16 (0.15-0.16)	<0.001
Received booster	0.33 (0.32-0.34)	<0.001
Background positivity rate	1.29 (1.28-1.29)	<0.001
High frequent testing profile	3.06 (2.99-3.12)	<0.001

B) factors associated with breakthrough infection among fully vaccinated persons when censoring at time of booster administration (time till infection, or otherwise till administration of booster or end of follow-up or death)

Factor	Hazard ratio (95%)	p-value
Age (per 10 years increase)	0.87 (0.86-0.87)	<0.001
Male sex	0.98 (0.97-0.99)	<0.001
Brand primary vaccine (ref: BNT162b2)		
mRNA-1273	0.68 (0.67-0.69)	<0.001
ChAdOx1	1.69 (1.68-1.71)	<0.001
Ad26.COV2.S	1.60 (1.58-1.62)	<0.001
Healthcare worker	0.59 (0.58-0.60)	<0.001
Prior COVID-19 infection	0.23 (0.22-0.23)	<0.001
Background positivity rate	1.38 (1.37-1.38)	<0.001
High frequent testing profile	4.06 (4.01-4.12)	<0.001

C) factors associated with breakthrough infection among boosted persons (time since booster dose till infection, or otherwise till end of follow-up or death)

Factor	Hazard ratio (95%)	p-value
Age (per 10 years increase)	0.84 (0.82-0.86)	<0.001
Male sex	1.12 (1.06-1.18)	<0.001
Brand primary vaccine (ref: BNT162b2)		
mRNA-1273	0.66 (0.59-0.74)	<0.001
ChAdOx1	0.91 (0.85-0.98)	0.011
Ad26.COV2.S	1.02 (0.86-1.21)	0.808
Healthcare worker	1.02 (0.92-1.12)	0.749
Prior COVID-19 infection	0.41 (0.36-0.47)	<0.001
Background positivity rate	1.01 (0.99-1.03)	0.422
High frequent testing profile	4.59 (4.13-5.10)	<0.001

A

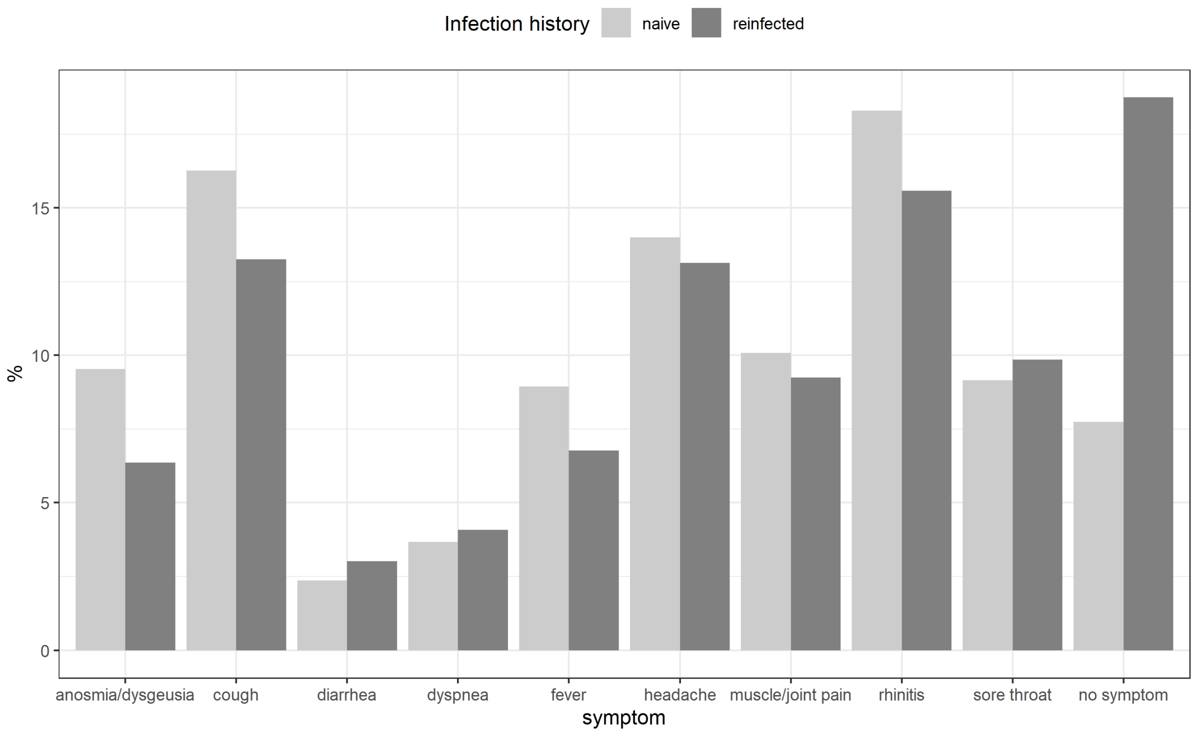

B

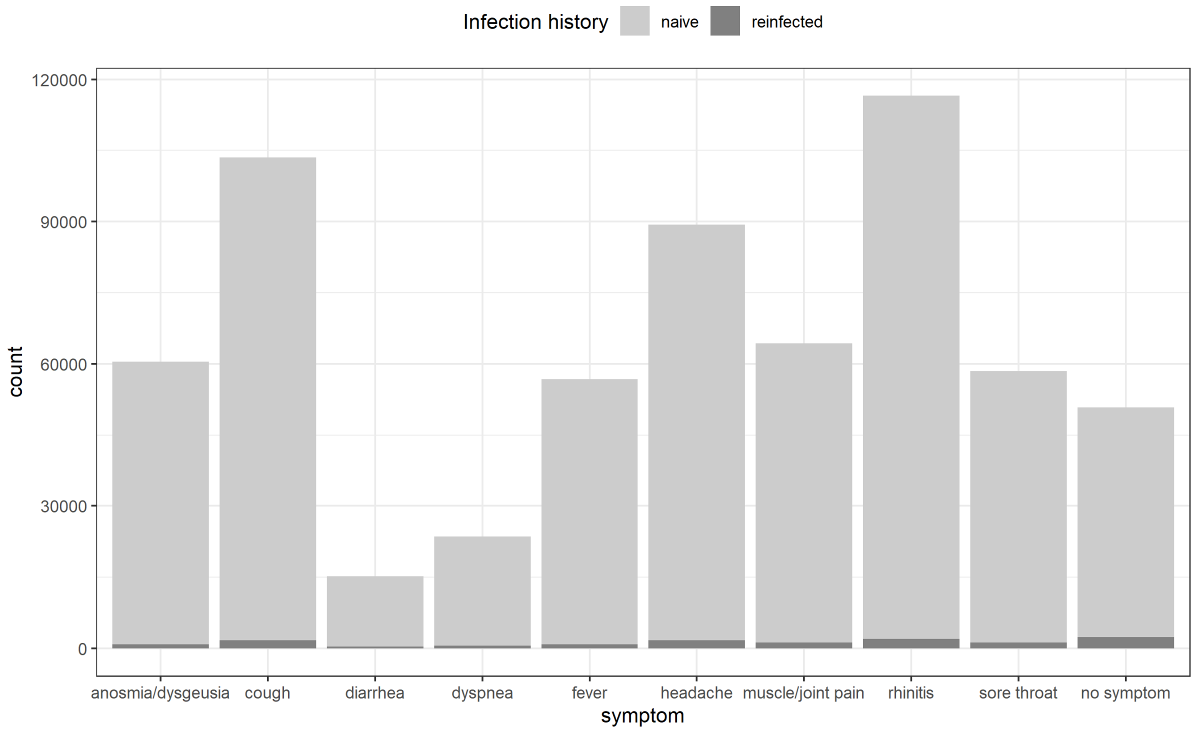

Figure S4: (A) Percentages and **(B)** counts of each type of symptom or of having no symptoms among persons with a breakthrough infection, grouped by having had a prior COVID-19 infection before vaccination (reinfected) or not (naïve).

Persons with a breakthrough infection and having available data on symptoms were taken into account for this analysis.
